# TDP-43-proteinopathy at the crossroads of tauopathy: on copathology and current and prospective biomarkers

**DOI:** 10.3389/fncel.2025.1671419

**Published:** 2025-10-28

**Authors:** Abdul R. Nasir, Claire Delpirou Nouh

**Affiliations:** Behavioral Neurology Division, Department of Neurology, Oklahoma University Health Sciences Center, Oklahoma City, OK, United States

**Keywords:** copathology, TDP-43, tauopathies, neuroimaging, biomarkers

## Abstract

Though usually described as isolated models, neurodegenerative diseases exist in a significant proportion of cases as mixed pathologies, particularly in older adults. The presence of co-pathologies may influence phenotypes and progression, and the correct classification *in vivo* has proven to be challenging, particularly without proper biomarker panels. Recent breakthroughs in biomarkers, enabling earlier detection in Alzheimer’s disease and, more recently, in synuclein-related diseases, are promising as a first step toward the wider detection of all other abnormal proteins involved in neurodegenerative diseases. Over the past decade, the growing body of research on TDP-43 pathology has led to considering TDP-43 as a potential major contributor to the neurodegenerative process. TDP-43’s normal function is essential for neuronal survival and the regulation of RNA processing and cellular stress response; abnormal TDP-43 protein leads to altered cell function and survival. TDP-43 is notably the neuropathological hallmark of amyotrophic lateral sclerosis (ALS) as well as some form of frontotemporolobar degeneration (FTLD). Tauopathies, divided in primary or secondary tauopathies cover other forms of FTLD including Pick disease (PiD), corticobasal degeneration (CBD), progressive supranuclear palsy (PSP) but also non-FTLD diseases like Alzheimer’s disease (AD) which can be classified as secondary tauopathy. As the importance of copathology is more and more recognized, TDP-43 is also frequently observed in conjunction with other proteinopathies, possibly with a synergistic or additive effect, although the exact mechanism is still unclear. In Alzheimer’s disease, the limbic predominant age-related TDP-43 encephalopathy neuropathologic change (LATE-NC) co-occurrence with Alzheimer’s disease neuropathologic changes (ADNC) lead to a more rapid course. Although there are currently no approved and validated biomarkers for its early detection, several promising tools, including neuroimaging and biofluid biomarkers, are under development, offering hope for the earlier detection of TDP-43 pathology *in vivo*. Accurate identification of the underlying proteinopathies and pathological processes could lead to better diagnosis and classification, more precise selection of clinical trial candidates, and ultimately, disease-specific tailored treatments.

## Introduction

Many neurodegenerative diseases are proteinopathies, characterized by the abnormal aggregation and accumulation of one or more misfolded proteins, which are thought to play a crucial role in their pathophysiology. With the advancement of biomarkers and the progress in understanding abnormal brain aging over recent years, multiple studies have demonstrated that most neurogenerative diseases involve more than one protein in complex interactions that contribute to neurodegeneration and symptoms (Rahimi and Kovacs, 2014). Since its first description in 2006 in frontotemporal lobar degeneration (FTLD) and amyotrophic lateral sclerosis (ALS), and over the past decade, the transactive response DNA binding protein of 43 kDa (TDP-43) has been identified as one of the key proteins associated with neurodegenerative diseases, along with amyloid, various strains of tau, and alpha-synuclein, among others (Neumann et al., 2006; Vanden Broeck et al., 2014). TDP-43 is a highly conserved and ubiquitously expressed RNA/DNA binding protein belonging to the hnRNP family of heterogeneous nuclear ribonucleoprotein (hnRNP) (Shenouda et al., 2022). It is an intranuclear protein encoded by the *TARDBP* gene, located on chromosome 1 (1p36.22), that can also shuttle to the cytoplasm depending on transcriptional needs. TDP-43 plays an important role in the functions of many cells, including RNA metabolism, mRNA transport, microRNA maturation, and cellular stress response (Prasad et al., 2019; de Boer et al., 2020). TDP-43 regulates its own expression via a complex mechanism and direct action on the TARDBP gene. Altered regulation or misdistribution of the protein will lead to altered cell function and survival, but the exact process is still poorly understood (Koyama et al., 2016).

Tau is another key protein whose dysfunction can lead to neurodegeneration. It is a microtubule-associated protein that stabilizes the neuronal cytoskeleton through this association and regulates axonal transport. It also plays a role in synaptic signaling and synaptic plasticity, as well as in axonal elongation and maturation, and is involved in RNA processing. Both TDP-43 and tau are RNA-binding proteins (RBP), involved in RNA regulation and mediating stress granule formation. Abnormal tau, including that caused by gene mutations or aberrant post-translational modifications— for example, hyperphosphorylation or N-glycosylation —is associated with a higher tendency to aggregate. These aggregates will form in the cytoplasm and alter normal neuronal function, while the absence of tau in the nucleus will, in turn, affect DNA and RNA processing and the maintenance of their integrity (Wang and Mandelkow, 2016; Koren et al., 2020; Samudra et al., 2023).

Abnormal conformation in TDP-43 can be observed in several neurodegenerative diseases, such as FTLD and ALS, where hyperphosphorylated and ubiquitinated TDP-43 accumulate as neuronal cytoplasmic inclusions identified during neuropathological examination (Neumann et al., 2007; Rutherford et al., 2008; Meneses et al., 2021), but also in Limbic-predominant Age-related TDP-43 Encephalopathy (LATE), an entity often found in the brains of older adults and present on average in approximately one-third of autopsies of individuals above 85 years old (Wilson et al., 2013; Nelson et al., 2022). The latter is defined by neuropathological changes in a limbic distribution with misfolded TDP-43 aggregates, referred to as LATE neuropathological change (LATE-NC). Misfolded proteins, including tau, are thought to be able to spread in a “prion-like” manner following the neuronal network, and TDP-43 is no exception either (Dugger and Dickson, 2017; Jo et al., 2020). TDP-43 pathology can co-occur in other proteinopathies, and with increasing interest in its possible role in tauopathies. Recent research suggests a potential interaction between both and possible synergistic effects; some studies suggest that TDP-43 pathology can exacerbate tau aggregation and seeding (Riku et al., 2022; Tomé et al., 2023; Tomé et al., 2024). In this review, we will examine the interaction between TDP-43 and tau and how this affects clinical assessment and diagnostics. We will discuss the ongoing development of TDP-43 biomarkers to facilitate a more precise identification of underlying pathology *in vivo*, which in turn will help optimize the development of therapeutic interventions.

### TDP-43 pathology

TDP-43 is a 414 amino acid protein with four domains, including a N-terminal domain, two RNA recognition motif domains (RRM1 and RRM2), and a C-terminal low-complexity domain (LCD) (Jiang et al., 2017). It also contains a nuclear localization signal (NLS) and a nuclear export signal (NES) that shuttles TDP-43 between the nucleus and the cytoplasm (Winton et al., 2008). The N-terminal domain mediates the formation of homodimers and oligomers (Chang et al., 2012) that participate in TDP-43’s physiological functioning and contains a sequence important for its transportation to the nucleus. The N-terminal domain may participate in TDP-43’s splicing function and protect it from forming cytoplasmic inclusions (Jiang et al., 2017). The RRMs are critical for its binding to RNA/DNA and exerting its role on mRNA, as well as forming ribonucleotide granules. The C-terminal low-complexity domain contains a glycine-rich region and a Glutamine/Asparagine (Q/N)-rich domain (Carrasco et al., 2023). This LCD prion-like domain (PrLD) mediates protein-protein interaction with other splicing factors, including heterogeneous nuclear ribonucleoprotein A1 (hnRNPA1), hnRNPA2B1, and fused in sarcoma (FUS) and is essential to regulate the splicing of some mRNA transcripts (Harrison and Shorter, 2017). PrLD is also important to the recruitment of TDP-43 into the formation of stress granules which are cytosolic structures that form transiently after cells are exposed to an environmental stress (Bentmann et al., 2012). C-terminal glycine-rich region regulates protein solubility. Most disease-related TDP-43 mutations are found in the LCD (Johnson et al., 2008; Corbet et al., 2021). PrLD seems to be participating in the aggregation process, and its deletion could suppress neurotoxicity (Ash et al., 2010). The LCD domain participates in the process of forming TDP-43 lipid droplets, known as liquid-liquid phase separation (LLPS), and perturbation of this phase formation may lead to pathological aggregation and dysfunction. However, this is still largely poorly understood (Babinchak et al., 2019; Corbet et al., 2021; Babinchak and Surewicz, 2023).

TDP-43 is an essential, highly conserved, and ubiquitously expressed RNA-binding protein involved in multiple steps of RNA processing, including transcription, translation, splicing, and stabilization and is encoded by the *TARDBP* gene on chromosome 1 (Ou et al., 1995; Cohen et al., 2011). It is a nuclear transcription factor regulating numerous genes (Buratti and Baralle, 2010). TDP-43 in physiological context is mostly present in the nucleus of the neurons but can also be found in oligomeric state in the cytoplasm (Kellett et al., 2025). The processus leading to mislocalization remains unclear. Mislocalized TDP-43 forms misfolded insoluble aggregates, some hyperphosphorylated and ubiquitinated, called “inclusion bodies” in the neuronal cytoplasm, as well as in nuclei and cell processes (neurites) of neurons and in oligodendroglia and astrocytes (de Boer et al., 2020). In 2006, TDP-43 was discovered as the major protein present in the ubiquitinated inclusions found in ALS (Lou Gehrig’s disease) (Arai et al., 2006) and has since lead to the discovery of its association with many neurodegenerative diseases and trial to understand it’s place in the degeneration cascade.

TDP-43 proteinopathy refers to a broad group of neurodegenerative processes in which one of the primary types of misfolded protein accumulation leading to typical inclusions found on neuropathological examination is TDP-43 (Liao et al., 2022). It can be divided into primary TDP-43 proteinopathies, referring to the disease driven primarily by TDP-43, which include FTLD-TDP, FTLD-ALS, for which TDP-43 is a pathological hallmark (Neumann et al., 2006; Kabashi et al., 2008; Sreedharan et al., 2008), and the Limbic-predominant Age-related TDP-43 Encephalopathy (LATE), which will be reviewed further later in this review. Perry syndrome is another rare form of TDP-43 proteinopathy, highlighting the broad spectrum of disorders associated with this proteinopathy (Wider et al., 2009; Mishima et al., 2018), and is an autosomal dominant neurodegenerative disease caused by a mutation in the dynactin 1 (*DCTN1)* gene on chromosome 2p13.1 that results in TDP-43 pathology (Ueda et al., 2024). It is clinically defined by neuropsychiatric features, including apathy that can be the initial symptom, severe depression, a symmetrical Parkinsonism usually poorly or transiently responsive to L-DOPA, significant weight loss, and central hypoventilation with respiratory failure being the most frequent cause of death (Perry et al., 1975; Dulski et al., 2021; Tsuboi et al., 2021). Mutation or downregulation of dynactin 1 has also been associated with sporadic or familial ALS (Laird et al., 2008). Perturbation of the microtubule-associated motor protein complex dynactin leads to dysfunction and dysregulation in stress granule disassembly in stressed cells, resulting in TDP-43 cytoplasmic accumulation (Ueda et al., 2024). Interestingly, tau and dynactin interact with each other, and the attachment of the dynactin complex to the microtubule is strengthened by tau, showing another connection between TDP-43 pathways and tau pathways (Magnani et al., 2007; Ueda et al., 2024).

Secondary TDP-43 proteinopathies refer to neurodegenerative diseases or non-neurodegenerative diseases in which TDP-43 plays a role and can be found associated with other proteins or pathological processes. This broader group include neurodegenerative diseases including Alzheimer’s disease, progressive supranuclear palsy (PSP), corticobasal degeneration (CBD), Parkinson’s disease (PD) (Chanson et al., 2010), multiple system atrophy (MSA) (Koga et al., 2018), Lewy body disease (Uchino et al., 2015), Huntington’s disease (HD) (Davidson et al., 2009), Primary age related tauopathy (PART) but also non-neurodegenerative disease like chronic traumatic encephalopathy (CTE) (McKee et al., 2010; McKee et al., 2015; Heyburn et al., 2019a; Heyburn et al., 2019b), brain tumors (Lin et al., 2017), or post-infectious or post-toxin exposure like in Parkinson-Guam dementia syndrome (Hasegawa et al., 2007; Kawakami et al., 2019; Ke et al., 2023; Rahic et al., 2023).

Cellular stress, including that related to toxin exposure, dysimmunity, and inflammation, may lead to TDP-43 dysfunctions and aggregation (Thammisetty et al., 2018; Bright et al., 2021; Masrori et al., 2022; Garamszegi et al., 2024). Genetic mutations beyond those in the TARDBP gene have also been linked to TDP-43 pathology, particularly C9Orf72 and GNR, and can serve as a common ground for some of these processes (Pickford et al., 2011; O’Rourke et al., 2016; Pottier et al., 2019; Kahriman et al., 2023). Both C9Orf72 and GNR genes are also associated with tauopathies.

Frontotemporal lobar degeneration (FTLD) refers to a clinicopathologic and genetically heterogeneous group of pathologies manifested by several and sometimes overlapping clinical syndromes that span from cognitive and behavioral symptoms like in Pick’s disease and behavioral variant FTD (bvFTD), to more language predominant symptoms in semantic primary progressive aphasia (svPPA) and nonfluent variant primary progressive aphasia (nfPPA) to include motor and movement symptoms in corticobasal degeneration (CBD), progressive supranuclear palsy (PSP) and amyotrophic lateral sclerosis (ALS) (Forman et al., 2007; Rabinovici et al., 2010; Younes and Miller, 2020; Neumann et al., 2021). At the neuropathological level, FTLD-TDP represents the most frequent underlying pathology in 45%–50% of cases, followed closely by FTLD-tau (40%–45%), then FTLD-FUS (5%–10%), and finally by other pathologies (Mackenzie et al., 2010). FTLD-TDP underlying pathology is widespread in the neocortex, hippocampus, and subcortical areas (Neumann et al., 2021). FTLD-TDP is itself divided into five major histological subtypes, categorized by the type of inclusions (designated as A to E), morphology, anatomical distribution, and cellular location. FTLD-tau is itself sub-classified according to the underlying strain of tau, which includes 3-repeat (3R) tau inclusion like found in Pick’s disease (PiD), 4-repeat (4R) tau pathologies-[CBD, PSP, aging-related tau astrogliopathy (ARTAG), Argyrophilic grain disease (AGD), and globular glial tauopathy (GGT)- or with both three and 4-repeat tau forms (3R/4R) as in PART and tangle only dementia (ToD) (Mackenzie et al., 2011; Neumann et al., 2021; Nilaver and Urbanski, 2023).

ALS is the most common adult-onset motoneuron disease characterized by an upper and lower motoneuron degeneration, leading to rapidly progressive paresis, which can lead to death in 2–4 years on average (Hobson and McDermott, 2016). Some behavioral and cognitive changes are frequent in as many as 50% of patients, while 5%–25% may meet criteria for clinical frontotemporal dementia (FTD). Up to 35% FTLD pathology is found in ALS autopsy series (Cividini et al., 2022). Upon neuropathological examination at autopsy, the pathognomonic finding of abnormal inclusion bodies is found in the cytoplasm of motor neurons. These inclusions are made up in more than 90% of cases of mislocalized and aggregated TDP-43 (Suk and Rousseaux, 2020). FTD-ALS cognitive symptoms can be similar to the behavioral variant FTD (BvFTD), while it can also present with a language variant, semantic variant in one third of FTD-ALS (Tan et al., 2019). Several other processes can be associated with TDP-43, including Hippocampal sclerosis (HS) of aging (Nelson et al., 2013; Nag et al., 2015; Nho et al., 2016; Cykowski et al., 2017; Nelson et al., 2019; Nelson et al., 2024).

All these neurodegenerative diseases are associated with the abnormal neuronal and glial accumulation of misfolded proteins; however, it remains unclear whether the pathological process results from a gain-of-function, loss-of-function, or both (Gendron and Petrucelli, 2009; Polymenidou et al., 2011; Wang and Mandelkow, 2016; de Boer et al., 2020; Ezzat et al., 2023). In the case of TDP-43, the abnormal trafficking of endogenous TDP-43 between the nucleus and the cytoplasm appears to lead to the formation of aggregates, including neuronal cytoplasmic inclusions (NCIs), neuronal intranuclear inclusions (NIIs), and/or dystrophic neurites (DNs), which collectively represent TDP-43 pathology (McAleese et al., 2017). As TDP-43 proteinopathy can be associated with many other neurodegenerative diseases, particularly tauopathies, considerable interest has grown in recent years to understand its role better, develop biomarkers to help recognize it outside of neuropathology/autopsy contexts, and ultimately provide future therapeutic approaches and clinical trials (Latimer and Liachko, 2021; Riku et al., 2022; VandeVrede et al., 2023).

### TDP-43 in mixte pathology with tau

Combined pathologies are increasingly recognized as an important field of investigation, as longitudinal studies have reported the co-occurrence of TDP-43 and tau. It becomes crucial to understand the various interactions at play in order to develop targeted therapies (Latimer and Liachko, 2021). TDP-43 has been shown to influence tau expression and protein levels, worsening tau aggregation and propagation (Gu et al., 2017; Riku et al., 2022; Tomé et al., 2023). However, as mentioned above, there is still considerable uncertainty regarding whether this is due to a loss of function, a gain of function, or both (Nelson et al., 2019; Wisse et al., 2025).

In the case of the primary TDP-43 proteinopathy LATE, LATE-NC can be found in one third of older adults above 85 years old and present with an amnestic syndrome like Alzheimer’s disease that is clinically indistinguishable from AD. It can cohabitate with other neurodegenerative diseases, and is very frequently found concomitantly with ADNC, with as many as up to 50% of cases in copathology in older adults (Nelson et al., 2019; Katsumata et al., 2022; Nelson et al., 2022). The presence of both pathologies, ADNC and LATE-NC, has been shown to worsen cognitive decline (Montine et al., 2022). When isolated, its course is usually slower, more limited to episodic memory, with some reports of behavioral manifestations that could be part of its picture (Brenowitz et al., 2014; Nag et al., 2018; Nelson et al., 2019; Liu et al., 2020; Ono et al., 2025). Neuropathology staging based on the anatomical progression of LATE-NC pathology was proposed by Nelson et al. (2019). Stage 1 involves TDP-43 pathology distribution limited to the amygdala, stage 2 involves the hippocampus, and stage 3 affects the amygdala, hippocampus, and the middle frontal gyrus (Nelson et al., 2019; Nelson et al., 2022). This is the most commonly used staging system, though a more detailed 5-stage system has been utilized for research purposes in some studies like the Religious Orders Study and Memory and Aging project (ROSMAP) (Nag and Schneider, 2023). More recently, Wolk et al. (2025) proposed criteria for the clinical diagnosis of LATE, distinguishing LATE-NC as a primary process not or minimally associated with ADNC and classifying it as possible or probable LATE, or when LATE-NC is found in a mixed pathology with AD. They defined core clinical criteria, including a primary amnestic syndrome with temporal-limbic memory loss and most other cognitive domains mostly spared. However, use of biomarkers remain critical and are required to help better distinguish both process, with the use of the MRI brain showing disproportional hippocampal atrophy as a marker of LATE-NC, as well as the presence or absence of AD biomarkers to classify the underlying pathology better and assess for AD; in case of positive AD biomarkers, additional testing is required using PET scan (tau PET and FDG-PET scan) (Wolk et al., 2025). The type of interaction between these two proteinopathies remains a subject of debate and research. Some hypotheses suggest a synergistic or additive effect, while others lean more toward a role in the timing of neurodegeneration progression. A concurrent progression between TDP-43 and AD stages, and particularly between tau and higher Braak stages, supports some interaction or synergy between the two. Moreover, the association extends beyond a parallel progression: the absence of TDP-43, even for the same burden of AD pathology, correlates with normal cognition, as noted in Josephs et al.’s (2014) study. Colocalization of both pathologies in the same neurons could support a common pathophysiological process (Montalbano et al., 2020). Tomé et al. (2021) also demonstrated that in AD, LATE-NC pathology was associated with an increased presence of NFT and phosphorylated tau (p-tau), as well as TDP-43, which increased p-tau aggregation and seeding (Nelson et al., 2022). *APOE E4* is associated with both an increased risk for AD and LATE-NC, suggesting some common pathophysiological pathways (de Flores et al., 2020; Dugan et al., 2021). TDP-43 pathology can also localize to the striatonigral system and present with parkinsonism and/or PSP-like syndrome (Murakami, 1999; Ono et al., 2025). In AD, it can be misinterpreted clinically for presence of Lewy Body copathology (Ono et al., 2025). In Ono et al.’s (2025) study, TDP-43 pathology correlated with reduced pigmented neuron density. As they used an antibody recognizing earlier stages of tau (pretangle and tangle), they found an association between tau and non-pigmented neuron density. Early tau pathology has been reported in the elderly above 90 years with Parkinsonism, even in the absence of AD or Lewy body pathology, which reinforces the importance of using markers for early tau to ensure that we capture the full spectrum of tau-related disease and co-pathology (Chu et al., 2024).

HS of aging (HS-aging) is frequently found in older adults as well, is associated with cognitive decline and dementia, and can present with an amnestic syndrome mimicking AD. It is defined by its neuropathological criteria with neuronal loss and gliosis in the hippocampi, out of proportion for an AD-only pathology. It is also frequently associated with ADNC, LATE-NC, and FTLD (Anderson et al., 2025). HS-aging is very frequently associated with TDP-43 pathology, leading to worse cognitive functioning and decline when they cohabitate (Nelson et al., 2013; Nag et al., 2015). Its diagnosis is mostly postmortem, with the typical neuropathological findings of neuronal loss and gliosis affecting principally the CA1 hippocampal subfield and subiculum. The cause of this selective vulnerability remains unclear, ranging from hypoxia, atherosclerotic disease, and being in a watershed area, to inflammatory, hyperexcitability, and excitotoxicity (Cole, 2007; Hatanpaa et al., 2014; Walker, 2015). TDP-43 presence was found to be associated with an increased likelihood of HS of aging, and inflammation may contribute (Nag et al., 2015).

In FTLD, the concomitant presence of TDP-43 and tau was previously considered rare, mainly due to an independent process or contextual circumstances, such as genetics or age (Robinson et al., 2014). However, mixed pathology is now being reported more frequently. Some limitations in sampling or the type of markers used may have prevented the identification of TDP-43 (Amador-Ortiz et al., 2007; Robinson et al., 2014; Kim et al., 2018; Koga et al., 2022). The choice of markers targeting the advanced, mature stage of tau in neurofibrillary tangles, such as the ghost tangle, may miss earlier and less mature stages of tau (Ono et al., 2025). In a neuropathology series of 201 autopsy-confirmed FTLD-TDP by Koga et al. (2022), 42% had concomitant ARTAG, 36% had PART, 22% had concurrent AGD, and finally 1% had pathology CBD. FTLD-TDP type A seems particularly at risk of being combined with tau pathology, and as much as in 50% of cases (Gefen et al., 2018). Interestingly, this group appeared to have a longer duration of disease and a longer lifespan, challenging the classic understanding that copathology accelerates the disease process and severity of symptoms. For FTLD driven by tau, the 4R tauopathy CBD appears to be the one showing the most frequent participation of TDP-43 in ∼16% of neuropathological cases in some series (Uryu et al., 2008; Uryu et al., 2008; Kim et al., 2018). In the Uryu et al. (2008) case series, which included 39 CBD pathological cases, 15.4% of the CBD cases exhibited some TDP-43 pathology, with 2 cases showing limited deposition in the dentate granule cells of the hippocampus, as well as in the entorhinal cortex. Four other cases had more diffuse aggregates observed in the temporal and frontal cortex, as well as the basal ganglia. There were no significant differences in age at death or disease duration between the TDP-43-positive and TDP-43-negative CBD groups in this specific study, although some other studies suggested that co-pathology could affect survival and disease duration (Uryu et al., 2008; Yamashita et al., 2014). TDP-43 and tau were sometimes colocalized, particularly in the frontal gray matter (Uryu et al., 2008). The distribution of TDP-43 pathology in the case by Kouri et al. (2013) was more unusual, differing from other previously reported cases of co-pathology in CBD, and was associated with both TDP-43 and tau pathology in the olivopontocerebellar system, suggesting a role in phenotypic presentation (Kouri et al., 2013). In FTLD-PSP, reports of the contribution of TDP-43 are more limited. Although initially thought to be more dependent on other pathologies like HS and AD, as cases with co-pathology were older in age and had higher Braak Neurofibrillary and Thal phases, some regions of vulnerability to PSP could also be affected by TDP-43. FTLD-PSP with concomitant tau and TDP-43 pathologies tend to have higher regional tau burden compared to TDP-43-negative ones, and a significant correlation between tau and TDP-43 burden was noted in the occipitotemporal gyrus, suggesting a potential interactive effect in this region (Yokota et al., 2010). TDP-43 and tau were frequently colocalized in the limbic system, particularly in the amygdala, where colocalization in the same neurons was observed (Kim et al., 2018). However, colocalization in the same neurons was not observed in some other regions, such as the hippocampal dentate gyrus, suggesting possible regional differences in pathophysiological mechanisms, as well as both independent and overlapping pathways (Yokota et al., 2010; Koga et al., 2017; Storey et al., 2017). Association could also be genetically predetermined, as it has been shown that PSP with TDP-43 had decreased expression of the TMEM106b homozygous minor allele gene, thought to be protective of TDP-43, compared to PSP without TDP-43 (Koga et al., 2017).

PART, previously called “Senile dementia of neurofibrillary type” or “tangle-predominant dementia,” is very frequently found in older brains, can mimic clinically amnestic AD but usually with milder symptoms that can still progress to dementia, and the progression is usually related to the tauopathic burden (Noda et al., 2006; Jellinger and Attems, 2007; Crary et al., 2014). Histologically, PART presents the same neurofibrillary tangles (NFT) as in AD, but without the presence of amyloid (Aβ) protein (“NFT+/Aβ-” brains) and with no involvement of the neocortex. The hippocampal tau burden also differs from that of classical AD, with the CA2 subsection being more involved than the CA1 subsection (Besser et al., 2017; Hickman et al., 2020). TDP-43 co-pathology in PART is usually less severe than in AD and may not significantly affect the clinical presentation (Josephs et al., 2017). Zhang et al. (2019) developed a staging system based on the TDP-43 dissemination sequence in PART, which is relatively similar to the one seen in AD, though more limited to the limbic system. Stage I has TDP-43 limited to the amygdala, spreading to the hippocampus in stage II, the neocortex in stage III, and finally to the putamen, pallidum, insular cortex, and the dentate gyrus of the hippocampus in stage IV (Zhang et al., 2019; Nag and Schneider, 2023).

We would like to include to this review the currently rarer but intriguing entities represented by the Guam Parkinsonism-Dementia Complex (G-PDC) and the amyotrophic lateral sclerosis-Parkinsonism-Dementia Complex (ALS-PDC), which may represent the same neurodegenerative disease with varying phenotypes. This now rare neurodegenerative disease endemic in Guam among the Chamorro people is characterized clinically by either primarily a Parkinsonian syndrome with dementia in G-PDC or a primarily motor presentation similar to classic sporadic ALS, sometimes associated with features of G-PDC either in the same individual or in the same family (Oyanagi, 2005; Verheijen et al., 2018). It was found to be secondary to exposure to a toxin from the seeds of cycad plants, which are used as food and in traditional medicine. The toxin may also precipitate genetic mutations, possibly via exposure during the prenatal or perinatal period (Spencer, 2022). It is characterized neuropathologically by both TDP-43 and tau copathology, and both pathologies are thought to contribute to the mechanisms of neurodegeneration (Geser et al., 2008). Other aggregates have been described associated with them, including Amyloid-β (Aβ) protein and alpha-synuclein (Condello et al., 2023). An inflammatory process resulting from exposure to the neurotoxin could be the primary basis for a common pathway leading to both TDP-43 and tau dysfunction. Several studies demonstrated a relationship between TDP-43 and immune-inflammatory pathways (Bright et al., 2021). Other studies have found a link between immunity and inflammation in the initiation of tau pathology and its progression (Johnson and Lukens, 2025). Cytoplasmic TDP-43 inclusions may be associated with defective RNA processing and other cellular disruptions, including mitochondrial dysfunction, nucleocytoplasmic transport, impaired endocytosis, and protein dysfunction (Verheijen et al., 2018). Glial cells can contain abnormal aggregates and may play a role in the pathophysiological process, as well as in the extracellular tau deposits, which increase microglial reactivity, a phenomenon also observed in other neurodegenerative diseases (Schwab et al., 1996; McGeer et al., 1997; Verheijen et al., 2018). The innate immune system and inflammation represent potential therapeutic targets; however, further research is needed to understand their relationship with neurodegenerative processes better (Bright et al., 2021). Similarly, TDP-43 can coexist with tau in Anti-IgLON5 disease, a neuroimmune disorder characterized by secondary tauopathy and neurodegeneration. TDP-43 and tau can either coexist or be found in distinct locations (Gelpi et al., 2016; Cagnin et al., 2017). One hypothesis is that the proinflammatory environment in neurons affected by the anti-IgLON5 antibody facilitates protein misfolding and neurodegeneration, which may lead to the accumulation of tau and secondary TDP-43; however, a synergy between both proteins is also a possibility (Gelpi et al., 2016). Traumatic brain injury (TBI) is associated with local inflammation in the area of the trauma, which can disseminate to other brain areas according to recent studies (Shi et al., 2019). Chronic Traumatic Encephalopathy (CTE) is a mixed 3R/4R tauopathy that mainly happens in the context of repeated head impacts (Cherry et al., 2020). Abnormal TDP-43 pathology is often observed on neuropathology and appears to progress with the stages of CTE and as tau pathology becomes more widespread, which suggests an association between the two processes (McKee et al., 2015; McKee et al., 2016; Heyburn et al., 2019b; Nicks et al., 2023; van Amerongen et al., 2023). A common denominator could potentially be a primary inflammation pathway leading to TDP-43 and tau dysregulation (Bright et al., 2021).

### The amygdala: a possible pivotal role in neurodegeneration

The frequent and early involvement of the amygdala in diverse proteinopathies raises the possibility of pathological synergies starting in the amygdala (Gonzalez-Rodriguez et al., 2023; Villar-Conde et al., 2023). The amygdala is a crucial brain structure in the anterior medial temporal lobe involved not only in emotions and behaviors, but also in memory and cognition (Avecillas-Chasin et al., 2023). A recent review from Stouffer et al. (2024) emphasizes the importance of the amygdala as an early involvement in AD, supported by early neuropsychiatric symptoms in some patients. The amygdala was also one of the sites with the earliest positivity on tau-PET in the Insel et al. (2020) study on AD, sometimes as early as 10 years prior to AD diagnosis. MRI brain and volumetric analysis studies have shown a relationship between areas, including amygdala atrophy, and neuropsychiatric symptoms in early disease, or as a predictor of AD diagnosis and dementia (Liu et al., 2010; Trzepacz et al., 2013). Amygdala subnuclei are also involved in FTLD to a diverse degree, depending on the underlying pathology (Bocchetta et al., 2019). The most affected are those with FTLD due to MAPT mutation carriers and in FTLD-TDP-43 type C (Bocchetta et al., 2021). It is also recognized in FTLD-ALS (Kawashima et al., 2001; Takeda et al., 2017). A recent study in ALS showed that intra-neuronal accumulation of TDP-43 in the amygdala correlated with behavioral changes in sporadic ALS (Rifai et al., 2024). Connectomics identifies the amygdala as a key hub in most neurodegenerative diseases. The propagation, also known as seeding of proteinopathies, typically spreads through a connected network, and the amygdala is a highly interconnected center, including the hippocampus and the prefrontal cortex (Ubeda-Bañon et al., 2020). Abnormal connectivity in the amygdala circuitry is happening early in AD and is identified as a possible marker of early disease (Kicik et al., 2025). Amygdala changes were also reported in cerebral small vessel disease (CSVD), with associated disrupted connectivity (Cheng et al., 2024). This association between vascular impairment, endothelial dysfunction and disruption of the amygdala-hippocampal circuitry may play a key role in neurodegeneration, as vascular disease is very frequently found on autopsy in neurodegenerative diseases (Cheng et al., 2024). Impaired perfusion, as well as local inflammation and metabolic dysfunction, could potentially drive or at least participate in triggering the neurodegenerative process in this location. Imaging studies looking at iron deposits as a marker of early neuronal damage found an association between iron deposition in the amygdala and vascular cognitive impairment (Cheng et al., 2024). The relationship could be bi-directional, and a recent study by Arribas et al. (2024) demonstrated the importance of endothelial TDP-43 for vascular integrity, highlighting that abnormal TDP-43 can also potentially lead to disruption of the blood-brain barrier and contribute to neuroinflammation. Its close anatomical proximity to the ependymal lining as well as the pia mater, particularly the ventromedial part, could play a role, as suggested by the presence of subependymal and subpial TDP-43 or tau. Atypical star-shaped TDP-43 inclusions have been recently identified, primarily in the subpial medial region of the amygdala, and they colocalize with tau in superagers (Geser et al., 2010; Kovacs et al., 2016; Nelson et al., 2018; Carlos et al., 2023a). It is hypothesized that this anatomical location close to the vasculature and CSF could increase exposure to extravasated plasma protein due to defects in blood-brain-barrier permeability, triggering pathways associated with neurodegeneration and proteinopathies. More research is needed to understand the mechanisms and timing better (Schultz et al., 2004; Lace et al., 2012). Appropriate sampling, including the amygdala, during neuropathology examinations, as well as the use of markers that allow the detection of tau at different stages of maturity (such as CP13 or PHF-1), and assessing for atypical TDP-43 inclusions, appears important to better assess the whole spectrum of co-pathology in the brain (Carlos et al., 2023a; Chu et al., 2024; Ono et al., 2025).

### Toward biomarkers for TDP-43

**FIGURE 1:**
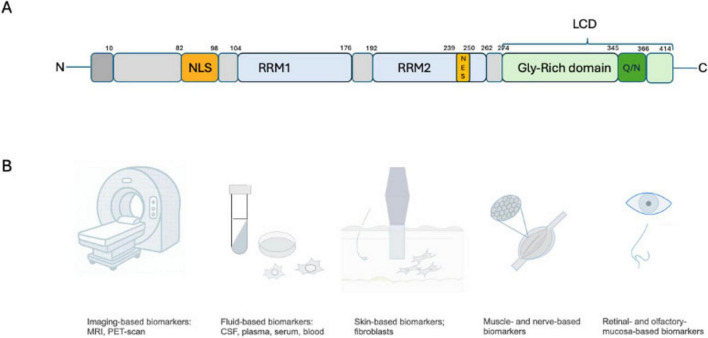
**(A)** TDP-43 protein contain a N-terminal domain, a nuclear localization sequence (NLS), two RNA recognition motifs (RRM1 and RRM2), a nuclear export sequence (NES), then the C-terminal domain/low complexity domain (LCD)/ prion-like domain with a glycine-rich domain and containing the Glutamine/Asparagine (Q/N) domain. **(B)** Potential target for biomarkers development, with most data coming from the neuroimaging field at that time, but with some promising development as well in biofluids and tissue-based markers.

The important role of TDP-43 in copathology and its potential synergistic effect on neurodegeneration, as reviewed above, raises the urgent need for biomarkers to facilitate more precise clinical diagnosis, which will help guide therapeutic approaches tailored to each underlying pathology and mechanism. Failing to identify copathology appropriately may lead to inappropriately interpreting clinical trial results, resulting in delays and setbacks (Figure 1).

#### MRI brain imaging

Neuroimaging is one of the most promising tools currently available to help identify TDP-43, either indirectly or directly. MRI can be used to assess neurodegeneration (N), and although it is not necessarily an early marker or a direct one, it still plays a key role in identifying atrophy patterns and neurodegeneration progression, helping with the diagnosis and staging process, and as a cue to assess for co-pathology (Jack et al., 2024; Youssef et al., 2025). Due to the lack of readily available *in vivo* molecular biomarkers for TDP-43 at this time, most data come from clinical-radiological and histological correlations, as cortical atrophy patterns seen on MRI correlate with progression and staging of TDP-43 pathology (Bejanin et al., 2019; Nelson et al., 2019). In all tauopathies, a greater volume loss is seen in the presence of co-pathology. In AD, the mesial temporal lobe (MTL) is usually the first affected. The presence of TDP-43 and HS coexisting with AD corresponds to additional disproportionate hippocampal volume loss on MRI brain compared to AD alone, which is a clue pointing toward multiple underlying proteinopathies (de Flores et al., 2020; Yu et al., 2020; Lyu et al., 2024; Wisse et al., 2025). Some previous longitudinal volumetric analyses have failed to correlate MTL volume with either amyloid or tau pathology, suggesting the presence of an additional factor. TDP-43 has been suggested here as a potential key actor in the potentiation of observed neurodegeneration. TDP-43 is thought to be associated with a greater degree of volume loss in AD, particularly when localized in the hippocampus, more so than when localized in the amygdala alone, and this independently of the presence of HS. The discrepancies between the amount of tau (for example, evaluated with tau-PET scan) compared to the degree of neurodegeneration are called the T-N mismatch (Carlos et al., 2023b), which correlated with non-AD pathology and particularly TDP-43 copathology as proven on neuropathology studies after autopsy (Josephs et al., 2017; Woodworth et al., 2022; Lyu et al., 2024). Tau was shown to correlate with a faster rate of atrophy early in the disease stage of AD, but had a lesser effect in the later stages of the disease (Josephs et al., 2017). Woodworth et al. (2022) also found a strong association between HS and hippocampal volume. Hippocampal subfield studies demonstrated a unique effect of TDP-43 with smaller CA1 and subiculum as well as inward deformation in bilateral CA1 and subiculum, and the most anterior portion of the left hippocampus. This deformation correlated with cognitive scores (Vos de Wael et al., 2018; Heywood et al., 2022). Pattern of MTL atrophy may differentiate AD with versus without TDP-43 pathology. de Flores et al. (2020) found a strong association between the anterior region of the MTL and TDP-43 (particularly in the entorhinal cortex and anterior hippocampus volumes). At the same time, tau was more associated with the posterior part of the hippocampus, and suggested using the ratio between the anterior hippocampus and the parahippocampal cortex that would serve as an early marker of TDP 43 beyond amygdala-only pathology in AD copathology (de Flores et al., 2020).

MRI brain scans enable volumetric analysis of other areas of interest, as well as the assessment of other possible confounders and co-pathologies. In one longitudinal MRI study, AD neuropathology was more closely associated with changes in ventricular volume than with hippocampal volumes, with CAA and vascular co-pathology also being potential contributors (Erten-Lyons et al., 2013). A more recent study with 3D-T1 3T-MRI showed that excessive amygdala volume loss could serve as a clinical biomarker for underlying TDP-43/LATE copathology (Wesseling et al., 2025). In Kim et al. (2018)’s study comparing the clinical and pathological presentations between the different subtypes of FTLD-TDP and FTLD-tau, the MRI brain showed that in FTLD-TDP as a primary disease with additional concurrent tau, there was more widespread atrophy compared to FTLD-TDP alone. Additionally, for primary FTLD-tau (CBD) with concomitant TDP-43, there was significant left asymmetrical atrophy, particularly in frontoparietal, hippocampal, striatum, and amygdala, when pure FTLD-tau (CBD) was associated with bilateral frontoparietal and basal ganglia atrophy, sparing the MTL (Kim et al., 2018; Buciuc et al., 2020; de Boer et al., 2020). More specific techniques, such as diffusion tensor imaging (DTI), are being studied. White matter (WM) changes are an important feature associated with the process of neurodegeneration and axonal loss. Structural MRI, as well as DTI, may allow for the identification of patterns that help distinguish them from what is usually attributed to small vessel disease. In ALS, Cheng et al. (2020) demonstrated macro and microstructural WM changes with alteration of the corticospinal tract and corpus callosum with increased mean diffusivity and decreased fractional anisotropy (FA), as well as decreased fiber density and bundle cross-section on Fixel-based analysis (FBA). A study by Maj et al. (2022) also suggested that using FA at the brainstem level and particularly in the pons could be a valuable biomarker for distinguishing ALS patients from healthy controls. Cortical tau load in AD has shown to be associated with worse WM burden (Brun and Englund, 1986; McAleese et al., 2015; Kantarci et al., 2017). Regional differences have been identified in AD, with temporal and parietal WM changes reported as correlating with the cortical axonal loss in AD, while frontal changes are a result of both small vessel disease and the AD degenerative process (Brun and Englund, 1986; McAleese et al., 2015). Patterns of atrophy have also been characterized in CBD and particularly in the premotor and supplemental areas (SMA) (Constantinides et al., 2019; Di Stasio et al., 2019). In PSP, the gray matter of the midbrain is involved, leading to the description of the “hummingbird sign” and “Mickey-mouse sign”; however, other regions can also be affected, including the superior cerebellar peduncles (SCP), the thalamus, and the frontal and motor areas (Albrecht et al., 2019; Lupascu et al., 2023). WM changes are also a key feature in PSP and CBD. FBA studies showed a specific pattern of bundle atrophy following axonal degeneration by quantifying fiber density and fiber bundle cross-section in 4R tauopathy and have been suggested as a better tool than DTI to assess *in vivo*, disease-specific, WM changes correlating with neuropathology findings associated with 4R tau spread and clinical symptoms, which could help monitor for disease progression. In PSP, involvement of the corpus callosum, as well as descending tracts from the primary motor cortex to the corona radiata, the internal capsule, thalamic radiation, and midbrain, is observed (Nguyen et al., 2021; Sakamoto et al., 2021; Uchida et al., 2023). FBA has also been used more recently in semantic variant dementia and is able to show a more comprehensive and specific map of WM changes than DTI, revealing early disruption in the anterior commissure, projections to the parahippocampal gyrus and amygdala, as well as parietal connection pathways in semantic dementia (Mandelli et al., 2025). FBA appears also able to differentiate changes due to presumed LATE from changes due to amnestic AD, and with alterations in the callosal fibers connecting the middle frontal gyri and of the cerebello-thalamo-cortical tracts in LATE, while involving more the callosal fibers connecting the superior frontal gyrus as well as temporo-limbic tracts in amnestic AD (Ahmadi et al., 2024; Lebrun et al., 2024; Vanderlinden et al., 2025). More studies will likely be conducted to expand our knowledge and the use of FBA in the future, enabling better assessment of TDP-43 copathology, not only in LATE and AD, but also in other tauopathies (Table 1).

**TABLE 1:** Summary of MRI findings in TDP-43 proteinopathies.

MRI	Findings	References
Hippocampus	- TDP-43 with smaller CA1 and subiculum; Inward deformation in bilateral CA1, subiculum and most anterior portions of hippocampus	Vos de Wael et al., 2018; Heywood et al., 2022
Mesial temporal lobe	- Anterior pattern of atrophy more correlating with TDP-43 (entorhinal cortex and anterior hippocampus volumes) - Posterior pattern of atrophy more associated with tau in AD - The ratio between the anterior hippocampus with the parahippocampal cortex as an early marker of TDP 43 beyond amygdala-only pathology in AD - Involvement of MTL in FTLD-tau suggest presence of TDP-43 copathology	de Flores et al., 2020; Kim et al., 2018
Amygdala	- Excessive volume loss suggestive for TDP-43/LATE co-pathology	Wesseling et al., 2025
Diffuse volume loss	- Worse widespread atrophy in FTLD-TDP when tau copathology	Kim et al., 2018; Buciuc et al., 2020; de Boer et al., 2020
White matter analysis	- White matter changes at the level of brainstem seen using fractional anisotropy (FA) in ALS may help distinguish from healthy control - Alteration of the corticospinal tract and corpus callosum in ALS using FA but also Fixel-based analysis (FBA) and correlating with functional scale in ALS - Temporal and parietal WM changes reported as correlating with the cortical axonal loss while frontal changes being a resultant of both small vessel disease and AD degenerative process - FBA showing early disruption on the anterior commissure, projections to the parahippocampal gyrus and amygdala as well as parietal connection pathways in semantic variant dementia, - FBA in LATE vs AD: alterations in the callosal fibers connecting the middle frontal gyri and of the cerebello-thalamo-cortical tracts whereas in amnestic AD callosal fibers connecting the superior frontal gyrus as well as temporo-limbic tracts	Maj et al., 2022; Cheng et al., 2020; Brun and Englund, 1986; McAleese et al., 2015; Mandelli et al., 2025; Lebrun et al., 2024; Ahmadi et al., 2024; Vanderlinden et al., 2025

FA, fractional anisotropy; FBA, fixel-based analysis; FTLD, frontotemporal lobar degeneration; LATE, limbic-predominant age-related TDP-43 encephalopathy; MTL, mesial temporal lobe; WM, white matter.

#### Positron emission tomography scan (PET-scan) imaging

FDG-PET is a non-specific neuroimaging test used to assess patterns of glucose metabolism in the brain. Beyond initial visual assessment, a quantitative analysis using software enables a comparison of the subject to the brain atlas of normal controls, utilizing a z-score. This approach ultimately allows for assessing whether the area of hypometabolism matches a specific pattern described in a particular pathology, thereby increasing confidence in diagnosis and accuracy (Na et al., 2024). However, this is not a specific test and should always be integrated into the broader clinical and imaging context. A hypometabolism pattern affecting the posterior parietal and temporal lobes, including the posterior cingulate, is suggestive of AD pathology (Jagust et al., 2007). Stage et al. (2020) showed that, however, in late-onset non-AD dementia, both pronounced atrophy and hypometabolism predominate for the bilateral temporal and prefrontal cortices, extending to the parietal lobes in more advanced disease, which was concordant with the pattern seen in LATE, or TDP-43-HS. The lack of tau binding in the medial temporal lobes seemed to exclude PART (Stage et al., 2020). A temporolimbic FDG-PET pattern, thought to correlate with LATE-NC staging, was also reported in other studies, including the one by Grothe et al. (2023). A pattern of hypometabolism in the MTL and the orbitofrontal cortex with preserved inferior temporal cortex metabolism leading to a high inferior temporal/MTL ratio is suggestive of LATE-NC underlying pathology (Botha et al., 2018; Grothe et al., 2023; Corriveau-Lecavalier et al., 2024). An amyloid-positive PET scan is a useful tool to rule in the presence of Alzheimer’s pathology. In contrast, a negative amyloid PET scan can help rule it out, which can help assess or rule out co-pathology (Matsuda et al., 2022; Wolk et al., 2025).

Tau-PET is currently not widely available in clinics and is primarily used in research. It could play a role not only from a diagnosis standpoint and identifying tau pathology, but also for staging purposes. The diversity and multiple strains of tau, which are also broadly divided into AD-tau and non-AD-tau, make finding tau ligands challenging (Choi et al., 2018; Schöll et al., 2019; Buchholz and Zempel, 2024). Moreover, tau is located both extra- and intracellularly, adding to the challenge in the development of a proper molecule. First-generation Tau ligand Flortaucipir shows strong affinity for AD tau but low affinity and off-target binding for other tauopathies (Ossenkoppele et al., 2018). Several second-generation ligands are currently promising and have less off-target binding (Smith et al., 2020). [18F]-MK-6240 has higher selectivity and is specific mainly for tau associated with Alzheimer’s disease, and with less to no binding in non-AD tau (Malarte et al., 2021). [18F]PI-2620 has shown promise as a candidate for use in non-AD pathologies (Tezuka et al., 2021; Cassinelli Petersen et al., 2022). It revealed a distinct pattern of binding in amyloid and non-amyloid corticobasal syndrome, which could aid in differential diagnosis and the identification of 4R tauopathies (Palleis et al., 2021). Brendel et al.’s (2020) study, which used a dynamic acquisition protocol, showed moderate-to-high discriminative performance between PSP and controls with [(18)F]PI-2620, characterized by increased uptake in the globus pallidus and subcortical regions associated with PSP (Yap et al., 2021). [18F]PI-2620 is currently in phase 3 of clinical development for the detection of tau in AD, as well as in 4R tauopathy like PSP and CBD, and received a fast track designation from the FDA. The study will evaluate cognitively normal seniors, as well as their ability to distinguish AD or FTLD-tau from FTLD-TDP, and assess their association with phenotypical features.^1^ [18F]OXD-2314 is another ligand showing promise in non-AD tau, pending further evaluation in patient populations of non-AD tauopathies (Lindberg et al., 2024). A negative tau PET scan, combined with suggestive FDG-PET findings for temporal-limbic hypometabolism, can suggest TDP-43 pathology (Botha et al., 2018; Stage et al., 2020).

Direct detection of TDP-43 aggregates by PET holds promises for a more accurate diagnosis, patient stratification, and assessment of therapeutic efficacy in clinical trials. Current research to identify the best candidate is ongoing. Some promising candidates have been reported, particularly the [18F]ACI-19278 tracer, which could become the first TDP-43 PET scan tracer in the future. This tracer showed high affinity for human brain-derived TDP-43 and appeared to be able to differentiate FTLD-TDP type A and B from controls in samples. It did not show off-target binding and was highly selective for TDP-43 (Seredenina et al., 2023). Seredenina et al. (2023) also reported that it quickly and efficiently crossed the blood-brain barrier and had a fast and complete washout, which limits the risk for a non-specific background signal. All these characteristics are promising, and the product is currently in an early-phase 1 clinical trial, with study completion estimated for late 2026.

#### TDP-43 biomarkers beyond neuroimaging

The development of fluid biomarkers for TDP-43 is ongoing, but has proved to be challenging; however, recent advancements using either antibody-based approach or proteomics show promise. Several issues remain, including the risk of binding to both the pathological and physiological forms of TDP-43, as well as variations in detected levels depending on solubility and sample origin. TDP-43 is a widely expressed protein, and its levels may not be explicitly related to CNS damage, but rather to damage in other organs (Cordts et al., 2023; López-Carbonero et al., 2024). Katisko et al. (2022)’s study utilized the Simoa^®^ sandwich enzyme-linked immunosorbent assay (ELISA) kit for TDP-43 to measure soluble TDP-43 in serum. Their study revealed a significant difference, with slightly decreased TDP-43 levels in FTD-TDP compared to FTD-tau and healthy controls (Katisko et al., 2022). Ren et al. (2021) also used a sandwich ELISA kit for TDP-43 and measured plasma and CSF TDP-43 levels, as well as phosphorylated TDP-43 (pTDP-43) levels, in ALS compared to healthy controls. The results showed that both TDP-43 and pTDP-43 were elevated in ALS and correlated well with CSF levels. The use of pTDP-43/TDP-43 in plasma helped differentiate between healthy controls and the ALS group and could be a good candidate as a biomarker in this context (Ren et al., 2021). The Multimer Detection System (MDS) platform, an atypical sandwich enzyme-linked immunosorbent assay (ELISA), was utilized by Jamerlan et al., (2023; 2025) and enabled the detection of increased oligomeric TDP-43 in the plasma of a small cohort of FTLD-semantic dementia. Another promising new technique is the proteomic platform using the Nucleic-Acid-Linked Immuno-Sandwich Assay (NULISA™), which utilizes oligonucleotide-conjugated antibodies to amplify signals from neurodegeneration-associated proteins, including those related to TDP-43 pathologies. Several studies have demonstrated its potential in detecting TDP-43 and pTDP-43 in plasma, suggesting its potential as a diagnostic tool in ALS and other TDP-43-related pathologies, including LATE-NC. However, the detection sensitivity remains suboptimal, and further studies are currently underway (Zeng et al., 2024; Thomas et al., 2025; Wang et al., 2025).

Another method currently evaluated uses the measurement of extracellular vesicles (EVs) in biofluids. EVs are cell-derived lipid nanoparticles that are released by cells into the extracellular environment, serving as transport vesicles that traffic macromolecules from the CNS to the cerebrospinal fluid (CSF) and blood. In LATE, EVs-TDP-43 derived from astrocyte-derived extracellular vesicles (ADEVs) was shown to be significantly increased in the plasma of individuals. This finding suggests that EVs-TDP-43 derived from neuronal and glial cells may serve as valuable diagnostic biomarkers in neurodegeneration, including LATE and potentially other TDP-43 proteinopathies. However, several questions and challenges remain, including the replicability and consistency of findings, the risk of potential artifacts from the EV enrichment material, and the time-consuming and often poorly reproducible methods of EV purification (Thompson et al., 2016; Sproviero et al., 2019; Pasetto et al., 2021; Winston et al., 2022; Dellar et al., 2025).

Another growing field of research for TDP-43 biomarkers focuses on the study of cryptic peptides. Abnormal TDP-43 affects its normal splicing function, leading to the inclusion of cryptic exons during transcription. This results in cryptic peptides from altered transcripts, which subsequently impair functions when not degraded by regulatory and monitoring pathways. Transcriptomic and proteomic approaches are currently being investigated to develop validated assays that assess cryptic peptides in biospecimens, thereby facilitating the detection of TDP-43 pathology, including co-pathology (Irwin et al., 2024; Seddighi et al., 2024).

Recent findings of high concentrations of TDP-43 in the cytosol of platelets have sparked interest in utilizing platelets as a potential biomarker (Wilhite et al., 2017; Luthi-Carter et al., 2024). Research focusing on ALS demonstrated increased levels of abnormal TDP-43 in platelets in the ALS group compared to healthy controls, and increased with disease duration (Hishizawa et al., 2019). It is hypothesized that abnormal TDP-43 could make its way from astrocytes to platelets via a permeable blood-brain barrier (Fang et al., 2014; Kopeikina and Ponomarev, 2021) or via platelet activation and release of platelet-activating factor (PAF) at the choroid plexus-blood-CSF barrier due to inflammation and leading to a leaky barrier (Čarna et al., 2023). However, much remains to be learned about utilizing platelets as a biomarker in copathology. Another approach by Quek et al. (2020) used ALS patient’s blood to generate monocyte-derived microglia (MDMi), which allowed the detection of TDP-43 and pTDP-43 cytoplasmic inclusions in ALS patients compared to healthy control. This model also helped demonstrating the mislocalization of TDP-43 in microglia in ALS patients (Quek et al., 2020; Quek et al., 2022). MDMi allows an easy sampling using blood collection, and shows promise as a screening tool in neurodegeneration and dementia beyond ALS (Banerjee et al., 2021; Quek and White, 2023).

The skin and the nervous system share the same ectodermal origin, leading to the concept of the skin-brain axis (Jameson et al., 2023; Kim et al., 2024), making skin an attractive candidate for assessing abnormal proteins and biomarkers in neurodegenerative diseases (Suzuki et al., 2010; Paré et al., 2015). There is already a fair amount of published work supporting the skin as a potentially accessible tissue for evaluating TDP-43 pathology (Sabatelli et al., 2015; Wang et al., 2015; Yang et al., 2015). Most studies comes from the ALS research and have shown a significant association between a higher amount of TDP-43 inclusion in ALS patients and a significantly higher amount of TDP-43 in the epidermis and dermis, as well as a higher amount of TDP-43 in the cytoplasm of dermal fibroblasts (Riancho et al., 2020; Romano et al., 2020; Rubio et al., 2022). Epidermal TDP-43 mRMA expression appears reduced in ALS patients, particularly in those with upper-limb onset (Abe et al., 2017). Ren et al. (2018) demonstrated the involvement of the peripheral and autonomic nervous systems in ALS patients, characterized by reduced intraepidermal nerve fiber density, as well as the deposition of TDP-43 and phosphorylated TDP-43 around autonomic nerve fibers. One study in a small cohort of sporadic ALS failed to demonstrate any specific changes in fibroblasts however (Codron et al., 2018), and more data remain needed in larger cohorts at different stages of the disease and particularly at an early stage as the amount of TDP-43 positive cells has been shown to be positively associated with the duration of the disease in ALS patients (Suzuki et al., 2010). Besides ALS, skin biopsy and fibroblast use have limited evidence in FTLD, which may be related to culture conditions and other limitations. However, fibroblasts may exhibit other markers of cellular stress that could be useful in identifying FTLD patients, and further research is ongoing (Riancho et al., 2020; Leskelä et al., 2021; Hoffmann and Haapasalo, 2022). Skin biopsy studies have shown promise in detecting tau using a tau seeding activity assay (tau-SAA), which exhibits a greater affinity for 4R tau than 3R tau, and notably demonstrates better accuracy in PSP (Vacchi et al., 2022; Dellarole et al., 2024; Martinez-Valbuena et al., 2024; Wang et al., 2024). Beyond the skin, muscle has also been investigated, mainly in neuromuscular diseases such as ALS. However, physiologically, TDP-43 is involved in the muscular regeneration process, and deposits are hypothesized to be more closely related to this process; further research is required (Ishikawa et al., 2012; Paré et al., 2015; Vogler et al., 2018; Liu et al., 2022). The olfactory mucosa is also studied, and TDP-43 aggregates using the TDP-43 seeding amplification assay (TDP43-SAA) have been shown to accurately distinguish TDP-43 pathology, pending further validation on a larger cohort (Fontana et al., 2024; Vizziello et al., 2025). Tau-SAA on the olfactory mucosa also has some limited positive data, but is considered invasive, increasing the risk of infections, and overall a less preferable option (Vacchi et al., 2025).

Retinal-based TDP-43 biomarkers are also being investigated, as some recent animal studies suggest early retinal changes in TDP-43 proteinopathies (Gao et al., 2024). However, there is still limited data in humans, primarily from autopsy reports (Glashutter et al., 2025). A small molecule tracer selectively binding TDP-43 in the retina is being evaluated through a phase 1/2 trial, the PROBE-trial, though no final results have been published yet, and research is ongoing (Glashutter et al., 2025). The use of nanotechnology, like the tau-fluorophore BT-1, a BODIPY-based probe and highly specific fluorescent ligand, is another promising technique that may expand our ability to evaluate for tau in the human retina and our ability for early detection of tauopathies (Soloperto et al., 2022; Barolo et al., 2024). The same techniques could potentially be used to develop TDP-43 probes.

In summary, the development of valid tools for the detection of TDP-43 in biofluids or other tissues proves to be challenging and is currently ongoing, including several other targets beyond plasma and CSF, with some data in skin and fibroblasts and pending larger studies, while retinal-based biomarkers using nanotechnology and TDP-43 or tau-probes may be promising, though also in need of further research and validation (Table 2).

**TABLE 2:** Summary of clinical trials on TDP-43 proteinopathy and biomarkers currently listed as recruiting/active in clinicaltrials.gov.

Trial ID	Type	Indication	Technique	Phase/study type	Status
NCT06891716	PET [18F] ACI-19626	ALS FTLD AD LATE	PET binding ligand for TDP-43	Early Phase I/Interventional	Recruiting
NCT05456503	PET FPI-2620	Non-amnestic AD FTLD-tau FTLD-TDP FTLD-genetic	PET imaging to differentiate tauopathy from TDP-43 proteinopathy	Phase III/Interventional	Recruiting
NCT05974579	PET 89Zr-DFO-AP-101	ALS	PET imaging study to detect misfolded SOD1	Phase I/Interventional	Active, not recruiting
NCT06735014	MRI Neurofilament light chain (NfL)	ALS	This imaging study evaluates the fiber density, fiber-cross section, orientation dispersion and cortical thickness on MRI and along use of plasma markers like NfL	Observational	Recruiting
NCT04960540	MRI	ALS	Imaging study for brain changes using fMRI, structural MRI and DTI	Observational	Unknown
NCT05764434	MRI	ALS	MRI study looking at imaging markers in ALS and more specifically changes in gray and white matter in the spinal cord.	Observational	Recruiting
NCT02567136	MRI	ALS	Imaging biomarkers using 3T and 7T MRI brain and DTI	Observational	recruiting
NCT02567136	MRI	ALS PLS	Imaging study using 3T and 7T MRI to detect early changes and biomarker of ALS and PLS	Observational	Active, not recruiting
NCT04691011	MRI	ALS	Imaging using 3T and 7T MRI at three different levels i.e., cerebral, medullary and muscular to determine early biomarkers in ALS	Interventional	Recruiting
NCT06528964	Skin biopsy	Neuro- degeneration	Skin biopsy study looking at alpha-synuclein, amyloid-beta, phosphorylated tau and TDP-43	Observational	Recruiting
NCT06490822	Skin biopsy	FTLD	Assess for TDP-43 and tau using Western blot and qPCR to determine level of expression of both proteins	Observational	recruiting
NCT05309408	Fluid biomarkers	ALS	Serum, plasma, whole blood, CSF, urine, peripheral blood mononuclear cells, DNA, RNA	Observational	recruiting
NCT06083584	RNA sequencing from blood	ALS	Targeted RNA-Seq for Amyotrophic Lateral Sclerosis Diagnosis	Observational	Recruiting
NCT03233646	Retinal imaging	Aging/ neurodegeneration	Evaluating the Retinal and Choroidal Microvasculature and Structure Using Multimodal Retinal and Choroidal Imaging in Neurodegenerative Disease: The iMIND Research Study	Observational	Recruiting

## Conclusion

TDP-43 frequently cohabits, though to varying degrees, with other neurodegenerative diseases, including tauopathies, and is suspected to be a major contributor to the neurodegenerative process. Several arguments suggest potential additive or synergistic effects with other proteins, particularly with tau, although common pathways and pathophysiological processes leading to multiple proteinopathies are also considered. The development of accurate and validated neuroimaging and fluid or tissue biomarkers is ongoing and will be crucial in identifying TDP-43 pathology and co-pathology, which will enable more precise diagnosis and *in vivo* pathology classification, facilitating the more accurate selection of candidates for clinical trials and allowing for future targeted and tailored treatments.

## Footnotes

1
https://clinicaltrials.gov/
